# DNA mismatch repair system expression in salivary gland tumors: A Systematic Review

**DOI:** 10.4317/medoral.26647

**Published:** 2024-06-22

**Authors:** Geórgya Mayara Travasso Torres Alves, Raisa Jordana Geraldine Severino-Lazo, Gleyson Kleber do Amaral-Silva, Fabiana Moura da Motta Silveira, Marianne de Vasconcelos Carvalho

**Affiliations:** 1 Department of Oral and Maxillofacial Pathology, School of Dentistry, Post-Graduation Program in Dentistry, University of Pernambuco (UPE), Recife, Pernambuco, Brazil; 2 Integrated Anatomic Pathology Center-Hospital Universitário Oswaldo Cruz, Recife, Pernambuco, Brazil; 3 Department of Oral Pathology, School of Dentistry, Federal University of Mato Grosso do Sul (UFMS), Campo Grande, Mato Grosso do Sul, Brazil; 4 Department of Oral Medicine, School of Health, Faculty Pernambucana of Health (FPS), Recife, Pernambuco, Brazil; 5 Hospital Dentistry Center-Hospital Professor Fernando Figueira Institute of Integrative Medicine, Recife, Pernambuco, Brazil

## Abstract

**Background:**

The DNA mismatch repair (MMR) system serves as a sophisticated guardian of the precise functioning of the human genome. Dysregulation within this system is linked to the oncogenesis process. Reduced expression of MMR system proteins identified in salivary gland tumors (SGTs) suggests an increased risk of tumoral occurrence. This study aims to analyze the expression of MMR proteins in SGTs and discuss the relevance of this association to the development of these neoplasms.

**Material and Methods:**

This review was conducted following the PRISMA guidelines and was registered in PROSPERO (CRD42023465590). A comprehensive search of the PubMed/MEDLINE, Web of Science, Scopus, Embase, and ProQuest (non-peer reviewed platform) was performed to answer the question “Do DNA MMR system proteins exhibit expression in SGTs?”. The methodological quality of the selected studies was assessed using the JBI’s Critical Appraisal Tool.

**Results:**

A total of 142 patients with benign SGTs and 84 with malignant SGTs were included in this review. The literature analysis showed a noTable reduction in the expression of DNA MMR system proteins (hHMS2, hMLH1, hMSH3 and hMSH6) in the percentage of marked cells.

**Conclusions:**

The reduction in the expression of the DNA MMR system proteins suggests an interesting correlation with the development of malignant and benign SGTs. Nevertheless, further investigations are warranted to better clarify the precision of measuring biomarker protein expression.

** Key words:**Salivary gland neoplasms, salivary gland cancer, DNA mismatch repair.

## Introduction

Salivary gland tumors (SGTs) constitute a heterogeneous group of lesions characterized by morphological diversity and inherent biological behaviors, representing approximately 3% to 10% of neoplasms within the head and neck region (1,2). According to the World Health Organization’s (WHO) Classification of Head and Neck Tumors, both major and minor salivary glands exhibit a remarkable diversity of alterations in differentiation patterns and architectural changes. This results in an overall annual incidence, considering all SGTs, ranging from 0.4 to 13.5 cases per 100,000 inhabitants (3). Due to their complex clinicopathological features, accurate diagnosis can be challenging.

Understanding the molecular biology of SGTs is significant for differential diagnosis and appropriate clinical management (4,5). In the fifth edition of the WHO classification, molecular data and biomarker studies have become widely referenced for both malignant and benign salivary neoplasms. These studies elucidate tumor-specific genetic rearrangements and have proved to be an important direction in comprehending these variable tumors (3-5). In this context, the evaluation of various proteins expressed in these tumors may provide insights into oncogenesis, pathogenesis, and open new pathways for the development of target-specific therapies.

Precise DNA replication is essential for maintaining genomic integrity and transmitting genetic information accurately. Dysfunctions in the repair mechanisms, which safeguard cells against potential mutation burden, are associated with an increased risk of developing various tumors. The DNA mismatch repair (MMR) system acts as a sophisticated protector of the human genome, encoding a set of error-correcting proteins involved in the replication of genetic material, thus preventing mutations (6-7). This system comprises a cluster of genes whose main protein subunits work together: MutSα (hMSH2-hMSH6), MutSβ (hMSH2-hMSH3) and MutLα (hMLH1-hPMS2). These complexes operate at the initiation of the MMR pathway in the cell cycle by preferentially detecting base-pair mismatches, and major insertion/deletion mispairs loops, and subsequently executing the excision of these errors (6,8,9). Consequently, the role of this system demonstrates relevance and cannot be underestimated.

Despite the diagnostic diversity, the oncogenesis of SGTs remains poorly understood. However, the reduced expression of proteins encoded by the DNA MMR system has been identified in SGTs and is believed by some authors to be linked with the development of lesions. In contrast, conflicting data regarding the response of this system require further investigation. Hence, the aim of this systematic review of published studies is to assess the involvement of the DNA MMR system protein expression in SGTs and discuss the relevance of this association to the development of these neoplasms. Thereby, the insights acquired from the study of the expression of DNA MMR system proteins in SGTs assume particular significance.

## Material and Methods

- Protocol and Registration

The present article followed the Preferred Reporting Items for Systematic Reviews and Meta-Analyses (PRISMA) checklist (10) and was registered in the International Prospective Register of Systematic Reviews (PROSPERO) under the registration number CRD42023465590.

- Eligibility Criteria

This systematic review followed a question formulated based on the “population, exposure, comparison, outcome, and study design of studies” (PECOS) criteria. The research question was: “Do DNA MMR system proteins exhibit expression in SGTs?”. The inclusion criteria addressed observational studies (cohort or cross-sectional) that evaluated the expression of DNA MMR system in patients with benign and/or malignant SGTs, describing the methods employed to detect the protein subunits comprising the system. Exclusion criteria included case reports, reviews, theses, dissertations, book chapters, studies reported in animals, studies with an uncertain diagnosis of SGTs, and those that did not elucidate the detection method for the MMR system.

- Search Strategy

A comprehensive search in electronic databases including PubMed/MEDLINE, Web of Science, Scopus, Embase, and ProQuest platform (non-peer-reviewed literature) was performed. No restrictions were imposed based on language or date. The search strategy is detailed in Table 1. Additionally, hand searching was performed in the reference list of included studies and in specific international journals in the field. To select included studies, the titles and abstracts of the articles were reviewed. Duplicates were removed using the (Rayyan Management software). Any discrepancies in the selection process between the investigators were resolved by a third researcher through discussion to reach a consensus.

- Data Collection Process

One researcher (GMTTA) collected data from the included articles, while a second researcher (RJGSL) checked all extracted data. The collected variables included the author, year, country, sample size, gender, mean age, location of tumors, pathological diagnosis of SGTs, type of tumors, the DNA MMR biomarkers, and the amount of the expression of DNA MMR system proteins.

## Results

- Screening and Selection of the Papers

**Figure 1:**
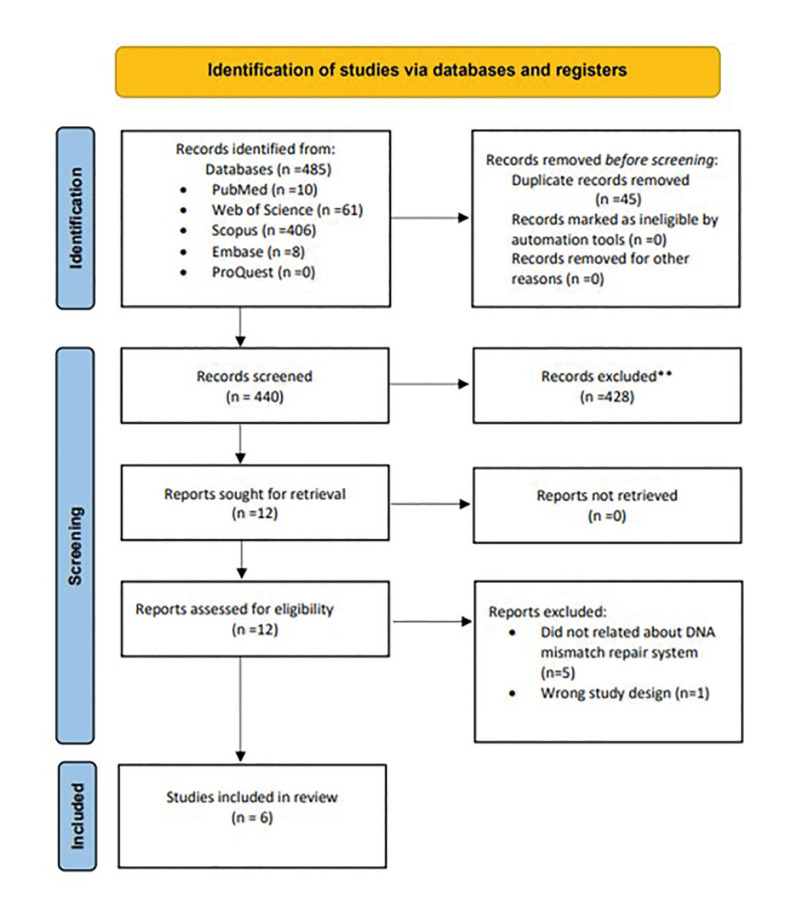
PRISMA flow diagram showing the study identification and selection process.

The comprehensive search, detailed in Fig. 1, initially identified a total of 485 relevant studies across various databases: 10 in PubMed/MEDLINE, 61 in Web of Science, 406 in Scopus, 8 in Embase, and 0 in ProQuest (non-peer-reviewed platform). After eliminating duplicates, 12 articles were selected for full-text analysis. Six studies met the inclusion criteria and underwent data extraction. Inter-rater agreement, assessed by Cohen’s Kappa coefficient during the article selection phase, demonstrated an “almost perfect agreement” between reviewers (kappa = 0.99). The methodological quality and risk of bias was assessed using the JBI Critical Appraisal Checklist for Analytical Cross-Sectional Studies (11). The JBI analysis is described in Table 2.

- Description of the Studies

A detailed overview of the included studies is presented in Table 3. This systematic review analyzed six cross-sectional studies published between 2001 and 2022. All the included studies evaluated the expression of the DNA MMR system in benign and malignant SGTs (12-17). The total sample comprised 236 tissue specimens from patients, with 50 males and 92 females. The mean age ranged from 36.29 to 60 years. The majority of tumors were located in the major salivary glands, with pleomorphic adenoma being the most frequently diagnosed benign pathological type in 104 cases (44.06%). Adenoid cystic carcinoma was the most frequently diagnosed malignant pathological type, representing 32 cases (13.52%). Benign lesions constituted the majority with 142 cases (60.15%), as opposed to malignant lesions evaluated in 84 cases (39.85%).

- DNA Mismatch Repair Proteins Expression

Regarding the percentage of cases, variable expression of the DNA MMR system was observed, ranging from 0% to 100% between benign and malignant SGTs. The range indicated a relevant heterogeneity between SGTs. However, considering the percentage of marked cells in SGTs, the expression of DNA MMR biomarkers showed noTable features. Tobón-Arroyave *et al*. (15) focusing only on benign SGTs, reported a underexpression of marked cells for biomarkers, ranging from 4.15 ± 3.05% to 7.27 ± 2.50% which aligned with the total mean. Castrilli *et al*. (12) showed important reduction levels of marked cell expression in benign STGs, varying from 14.0 ± 12.6% to 31.1 ± 22.6% which also showed a decreased expression reported by the total mean. Statistical analysis revealed a significant difference in hHMS2 protein expression among the benign lesion groups used in both studies (Mann-Whitney test, *p*=0.003). Similarly, significant difference was observed concerning hMLH1 protein expression (*p*=0.000). Benign SGTs showed reduced expression of DNA MMR system proteins. Soares *et al*. (14) focusing only on malignant SGTs, reported an expression ranging from 56.5% to 62.5%. However, they showed a reduction in the total mean expression in the labeled cells, from 29.25% to 45.25%. Castrilli *et al*. (12) comprising a wide range of malignant SGTs, showed a similar balanced reduction in the marked cells as emphasized by the total mean expression, from 56.1 ± 31.6% to 27.9 ± 26.0%. Significant differences were observed in hHMS2 and hMLH1 proteins comparing malignant lesions in both studies (*p*=0.002) and (*p*=0.000), respectively. Malignant SGTs also showed reduced expression of DNA MMR system proteins. Nevertheless, Amaral-Silva *et al*. (13) showed a decreased levels of marked cells expression in both malignant and benign SGTs, ranging from 4.27 ± 5.35% to 46.47 ± 18.06% and 1.73 ± 1.20% to 46.37 ± 22.35%, respectively. The total mean for hMSH3 (6.25 ± 6.95%) and hMSH6 (10.81 ± 6.45%) also reported a lower proportion of malignant SGTs compared to hMSH2 and hMLH1. The available data on DNA MMR system expression is summarized in Table 4. Detailed results of total mean expression in marked cells are presented in the Supplementary Material.

## Discussion

The DNA MMR system is widely recognized as an essential mechanism for genome stability, playing a pivotal role in repairing base-base or insertion-deletion errors during DNA replication (6,7). This process has a significant impact on mutagenic suppression in dividing cells (8,12-14,18,19). Overall, we found a reduced expression of proteins encoded by the DNA MMR system in the percentage of marked cells. This result suggests an interesting association with the development of these tumors since the uncorrected spontaneous mutation by the system induces an increase in cell proliferation and tumor invasion. Furthermore, increased risk through MMR deficient expression has been observed in important syndromic manifestations such as Lynch Syndrome, Muir-Torre Syndrome, and Turcot Syndrome (7,8,9,20).

Immunohistochemistry (IHC) has been a potent tool for scrutinizing the deficient protein expression of DNA MMR subunits in diverse tumors for more than two decades (20-22). All studies in this systematic review used this method to evaluate the DNA MMR proteins expression in SGTs (12-17). This review showed that the system expression of SGTs data was typically categorized into the positive percentage of marked cells and the positive percentage of cases. When, considering the percentage of cases without accounting for the percentage of cells marked by each subunit expressing this repair, implies the creation of a gap in the understanding of this phenomenon. Conversely, considering the percentage of marked cells may offer a more nuanced elucidation of the results by acknowledging through measurement of the individual contributions of MMR proteins in tumor cells (12-17).

Regarding the hHMS2 protein, a balanced reduced nuclear expression in cells within SGTs was identified for our analysis. This protein subunit is linked to the function of recognizing DNA damage (6,8,9). Interestingly, the analysis showed that the decreased expression of this protein is more noTable in benign SGTs. Castrilli *et al*. (12) showed that compared to malignant tumors, benign tumors exhibited a heightened loss of total mean expression of this protein, 31.1± 22.6%. In accordance, Tobón-Arroyave *et al*. (15) reported a substantial underexpression of marked cells for this biomarker in pleomorphic adenoma, 7.27 ± 2.50%. This could probably be explained that, since the DNA MMR system is involved in a wide range of activities linked to primary cell stability, reduced expression means equivalent loss of cell checkpoint control. The literature associates deficient expression of this protein with a higher risk of extracolonic tumors (18). However, dysfunctions in the expression of DNA MMR system proteins have been linked to Lynch Syndrome. This condition is characterized by an increased risk of several cancers affecting multiple anatomical regions, encompassing the head and neck. These related neoplasms include colorectal cancer, endometrial cancer, ovarian cancer, stomach cancer, small intestine cancer, urinary tract cancer, biliary tract cancer, brain tumors (typically glioblastoma/Turcot Syndrome), sebaceous adenomas, sebaceous adenocarcinomas (Muir-Torre Syndrome), keratoacanthomas, pancreatic cancer, and prostate cancer (20). This comprehensive scope underscores how deficiencies in DNA MMR protein expression can impact a variety of organs and tissues.

Similarly, examinations of the hMLH1 protein in SGTs exhibited relevant findings. In accordance with the available data, malignant and benign SGTs demonstrated expression reduction of marked cells for this biomarker. Tobón-Arroyave *et al*. (15) showed that underexpressed percentages in labeled cells for this biomarker (4.15 ±3.05%). Underlining this association with benign SGTs, Castrilli *et al*. (12) also demonstrated a decrease in total mean expression in benign SGTs (14.0 ± 12.6%). Interestingly, a noteworthy underexpression in hMLH1 and hHMS2 was directly reported in sebaceous adenocarcinoma associated with MTS, ranging from 28% to 2% respectively, highlighting this type of malignant SGTs development (14). This has already been mentioned in the WHO Classification of Head and Neck Tumors (3). The reduced expression of this protein correlates with the progression from preneoplastic lesions to oral squamous cell carcinoma and behavior of oral invasive malignancies (22). In addition, deficient MLH1 expression is associated with the development of colorectal cancer at a younger age and occurrence of metastasis in breast cancer cases (18).

Intriguingly, contradictory findings were observed for both hHMS2 and hMLH1 in warthin tumors, the second most common benign neoplasm of the salivary glands (1,2,23). Castrilli *et al*.'s (12) results showed total case negativity for the system aligned with their cell markings (0% expression), while Hunt (16) reported total positivity of cases (100% expression) in this tumor type. However, considering the system's working mechanism, these disparities could probably be explained by the fact that mutation's behavior can cause generalized or only areas of zonal defects in neoplastic tissues. Most notably, Amaral- Silva *et al*. (13) reported that marked cells of warthin tumors showed an interesting underexpression for the hMSH6 biomarker (1.73 ± 1.20%), ranging from 0.1 - 4.6%. Meanwhile, dysfunctions in both hMSH2 and hMLH1 expression is also correlated with higher levels of bone invasion, as well as the presence of metachronous neoplasms (22).

Recent investigations introduced a new dimension to the discussion by analyzing two previously unstudied DNA MMR system subunits, hMSH3 and hMSH6. Remarkably, malignant and benign SGTs presented a lower percentage of marked cells expressed for hMSH3, with their lowest expression represented by 4.27 ± 5.35% and 7.30 ± 3.41%, respectively (13). Amaral-Silva *et al*. (11) reported that malignant tumors showed an underexpression with a total mean of 6.25 ± 6.95% in cells marked for the activity of hMSH3 biomarker. Malignant SGTs exhibited a lower total mean than benign SGTs, suggesting a higher lack of hMSH3 expression. Deficient expression of hMSH3 protein is commonly observed in esophageal carcinoma - present in 91% of tumors compared to 76% in adjacent normal esophageal tissue (13). Similarly, the hMSH6 biomarker also demonstrated a significant underexpression in labeled cells in malignant (10.81 ± 6.45%) and benign SGTs (5.45 ± 5.05%). In addition, deficiency of hMSH6 expression is associated with colorectal cancer and genetic alterations in breast cancer, potentially impacting the response to immunotherapies (18,20).

In summary, this systematic review underscores the intricacy of the DNA MMR system protein expression in SGTs. However, it is essential to acknowledge some limitations of the studies. The scarcity of primary studies on this subject and the absence of studies with larger samples due to the unusual lesions hindered further statistical analysis, thereby limiting the scope of our results. Moreover, further investigations are warranted to better elucidate the precision of measuring protein expression in the DNA MMR system for SGTs and explore its implications. The protein expression presents the potential to contribute to a more robust understanding of the role of the DNA MMR system in salivary gland tumorigenesis, thereby providing valuable insights for clinical decision-making and potential target therapeutic interventions in the future, ultimately leading to better outcomes for patients.

## Conclusions

The analysis conducted in this systematic review suggests an interesting association between reduced expression of the DNA MMR system proteins and the development of malignant and benign SGTs.

## Figures and Tables

**Table 1:** Search strategy in each electronic database.

Electronic database	Search strategy	
PubMed	#1	((("Salivary Gland Neoplasms"[Mesh]) OR ("Salivary Glands, Minor"[Mesh])) OR ("Salivary Glands"[Mesh])) OR ("Salivary gland tumors")
	#2	((((((((((((((((((("DNA Mismatch Repair"[Mesh]) OR ("MutS DNA Mismatch-Binding Protein"[Mesh])) OR ("G-T mismatch-binding protein" [Supplementary Concept])) OR ("MutS Proteins"[Mesh])) OR ("MutS Homolog 3 Protein"[Mesh])) OR ("MutS Homolog 2 Protein"[Mesh])) OR ("MSH3 protein, human" [Supplementary Concept])) OR ("MSH2 protein, human" [Supplementary Concept])) OR ("MutL Proteins"[Mesh])) OR ("MutL Protein Homolog 1"[Mesh])) OR ("MLH1 protein, human" [Supplementary Concept])) OR ("Mismatch Repair Endonuclease PMS2")) OR ("MutS beta")) OR ("MutS alfa")) OR ("MutLa")) OR ("hMSH6")) OR ("hMSH2")) OR ("hMSH3")) OR ("hMLH1")) OR ("hPMS2")
	#3	#1 AND #2
Web of Science	#1	(((ALL= ("Salivary Gland Neoplasms")) OR ALL=("Salivary Glands, Minor")) OR ALL=("Salivary Glands")) OR ALL=("Salivary gland tumors")
	#2	((((((((((((((((((((ALL=("DNA Mismatch Repair'')) OR ALL=("MutS DNA Mismatch-Binding Protein")) OR ALL=("G-T mismatch-binding protein")) OR ALL=("MutS Proteins")) OR ALL=("MutS Homolog 3 Protein"')) OR ALL=(" MutS Homolog 2 Protein")) OR ALL=("MSH3 protein, human")) OR ALL=("MSH2 protein, human")) OR ALL=("MutL Proteins")) OR ALL=("MutL Protein Homolog 1"))
	#3	#1 AND #2
Scopus	#1	( ALL ( "salivary gland neoplasms" ) OR ALL ( "salivary glands, minor" ) OR ALL ( "salivary glands" ) OR ALL ( "salivary gland tumors" ) )
	#2	( ALL ( "dna mismatch repair" ) OR ALL ( "muts dna mismatch-binding protein" ) OR ALL ( "g-t mismatch-binding protein" ) OR ALL ( "muts proteins" ) OR ALL ( "muts homolog 3 protein" ) OR ALL ( "muts homolog 2 protein" ) OR ALL ( "msh3 protein, human" ) OR ALL ( "msh2 protein, human" ) OR ALL ( "mutl proteins" ) OR ALL ( "mutl protein homolog 1" ) OR ALL ( "mlh1 protein, human" ) OR ALL ( "mismatch repair endonuclease pms2" ) OR ALL ( "muts beta" ) OR ALL ( "muts alfa" ) OR ALL ( "mutla" ) OR ALL ( "hmsh6" ) OR ALL ( "hmsh2" ) OR ALL ( "hmsh3" ) OR ALL ( "hmlh1" ) OR ALL ( "hpms2" ) )
	#3	#1 AND #2
Embase	#1	‘salivary gland cancer'/exp OR ‘salivary gland'/exp OR ‘salivary gland disease'/exp OR ‘salivary gland tumor'/exp
	#2	‘mismatch repair'/exp OR ‘mismatch repair protein pms2'/exp OR ‘mismatch repair protein'/exp OR ‘protein msh3'/exp OR ‘mutl protein homolog 1'/exp OR ‘protein muts'/exp OR ‘protein mutl'/exp OR ‘dna mismatch repair protein msh2'/exp OR ‘hmsh6 gene'/exp
	#3	#1 AND #2
ProQuest	#1	noft("Salivary Gland Neoplasms" OR "Salivary Glands" OR " Salivary Gland Tumors")
	#2	noft("DNA Mismatch Repair" OR "MutS DNA Mismatch-Binding Protein" OR "MutS Proteins")
	#3	#1 AND #2

**Table 2:** JBI critical appraisal checklist for analytical cross-sectional studies.

Questions	Ohki *et al.* (2001)	Castrilli *et al.* (2002)	Hunt (2006)	Tobón-Arroyave *et al.* (2009)	Soares *et al.* (2018)	Amaral-Silva *et al.* (2022)
Were the criteria for inclusion? in the sample clearly defined?	Yes	Yes	Yes	Yes	Yes	Yes
Were the study subjects and the setting described in detail?'	Yes	Yes	Yes	Yes	Yes	Yes
Was the exposure measured in a valid and reliable way?	Yes	Yes	Yes	Yes	Yes	Yes
Were objective, standard criteria used for measurement of the condition?	Yes	Yes	Yes	Yes	Yes	Yes
Were confounding factors identified?	Yes	No	No	Yes	Yes	No
Were strategies to deal with confounding factors stated?	Yes	No	No	Yes	Yes	No
Were the outcomes meansured in a valid and reliable way?	Yes	Yes	Yes	Yes	Yes	Yes
Was appropriate statistical analysis used?	Yes	Yes	Not applicable	Yes	Yes	Yes

**Table 3:** Characteristics of included studies.

Author and year	Country	Sample size	Gender M/F	Mean age	Location of tumors	Pathological diagnosis	Type of tumors
Ohki *et al.* (2001)	Japan	14	NR	NR	Major	Pleomorphic Adenoma	Benign
		4				Warthin Tumor	Benign
		1				Acinic Cell Carcinoma	Malignant
		9				Adenoid Cystic Carcinoma	Malignant
		3				Mucoepidermoid Carcinoma	Malignant
		3				Carcinoma in Pleomorphic Adenoma	Malignant
Castrilli *et al.* (2002)	Italy	18	NR	NR	Major	Pleomorphic Adenoma	Benign
		5				Warthin Tumor	Benign
		4				Adenoid Cystic Carcinoma	Malignant
		4				Mucoepidermoid Carcinoma	Malignant
		1				Squamous Cell Carcinoma	Malignant
		3				Acinic Cell Adenocarcinoma	Malignant
		1				Basal Cell Adenocarcinoma	Malignant
		4				NOS	Malignant
		1				Polymorphous Low-grade Adenocarcinoma	Malignant
		2				Malignant Mixed Tumor	Malignant
		NR				Non-neoplastic gland	Normal
Hunt (2006)	USA	12	4/8	60	Major	Warthin Tumor	Benign
Tobón-Arroyave *et al.* (2009)	Colombia	35	9/26	36.29	Minor	Pleomorphic Adenoma	Benign
Soares *et al.* (2018)	Brazil	9	3/6	62.1	Major	Sebaceous Adenocarcinoma	Malignant
		1	1/6			Sebaceous Adenocarcinoma associated with MT	
Amaral-Silva *et al.* (2022)	Brazil	37	33/52	44	Major and minor	Pleomorphic Adenoma	Benign
		17		55		Warthin Tumor	Benign
		19		49		Adenoid Cystic Carcinoma	Malignant
		19		50		Mucoepidermoid Carcinoma	Malignant
		10		NR		Normal Salivary Gland	Normal

M: Male; F: Female; NR: Not Reported; NOS: Not Otherwise Specified Adenocarcinoma; MTS:Muir-Torre Syndrome.

**Table 4:** Assessment of expression of DNA mismatch repair system.

Author and year	Pathological diagnosis	Sample size	DNA mismatch biomarker (antibody)	Clone	Positive percentage of cases	Positive percentage of marked cells
Benign Tumors						
Ohki *et al.* (2001)	Pleomorphic Adenoma	14	hMSH2	NR	100% (14/14)	NR
	Warthin Tumor	4	hMSH2	NR	100% (4/4)	NR
Castrilli *et al.* (2002)	Pleomorphic Adenoma	18	hMSH2	FE11	100% (18/18)	31.1 ± 22.6 % range 6-78%
	Pleomorphic Adenoma	18	hMLH1	G168-728	72.22% (13/18)	14.0 ± 12.6 % range 5-51%
	Warthin Tumor	5	hMSH2	FE11	0% (0/5)	0%
	Warthin Tumor	5	hMLH1	G168-728	0% (0/5)	0%
	Non- neoplastic gland	NR	hMSH2	FE11	NR	NR
	Non- neoplastic gland	NR	hMLH1	G168-728	NR	NR
Hunt (2006)	Warthin Tumor	12	hMSH2	NR	100% (12/12)	NR
	Warthin Tumor	12	hMLH1	G175- 405	100% (12/12)	NR
Tobón-Arroyave *et al.* (2009)	Pleomorphic Adenoma	35	hMSH2	FE11	88.57 % (31/35)	7.27 ± 2.5%
	Pleomorphic Adenoma	35	hMLH1	Clone 14	82.85% (29/35)	4.15 ± 3.05%
Amaral-Silva *et al.* (2022)	Pleomorphic Adenoma	37	hMSH2	Polyclonal	100%(37/37)	46.37 ± 22.35% range 11 - 82.6%
	Pleomorphic Adenoma	37	hMSH3	Polyclonal	NR	14.53 ± 12.36% range 1.2 - 53.3%
	Pleomorphic Adenoma	37	hMSH6	Polyclonal	NR	9.17 ± 8.91% range 0.4 - 30.6%
	Warthin Tumor	17	hMSH2	Polyclonal	100%(17/17)	28.10 ± 7.63% range 16 - 43%
	Warthin Tumor	17	hMSH3	Polyclonal	NR	7.30 ± 3.41% range 2.4 - 15%
	Warthin Tumor	17	hMSH6	Polyclonal	NR	1.73 ± 1.20% range 0.1 - 4.6%
	Normal Salivary Gland	10	hMSH2	Polyclonal	100%(10/10)	47.35 ± 24.57% range 8.4 - 79.7%
	Normal Salivary Gland	10	hMSH3	Polyclonal	100%(17/17)	25.58 ± 20.74% range 1.5 - 53.3%
	Normal Salivary Gland	10	hMSH6	Polyclonal	100%(10/10)	42.38 ± 22.62% range 3.8 - 77.4%
Malignant Tumors						
Ohki *et al.* (2001)	Acinic Cell Carcinoma	1	hMSH2	NR	100%(1/1)	NR
	Adenoid Cystic Carcinoma	9	hMSH2	NR	100%(9/9)	NR
	Mucoepidermoid Carcinoma	3	hMSH2	NR	100%(3/3)	NR
	Carcinoma in Pleomorphic Adenoma	3	hMSH2	NR	100%(3/3)	NR
Castrilli *et al.* (2002)	Adenoid Cystic Carcinoma	4	hMSH2	FE11	100% (4/4)	42.38 ± 22.62% range 10 - 89%
	Adenoid Cystic Carcinoma	4	hMLH1	G168-728	100%(4/4)	26.5 ± 33.1% range 6 - 76%
	Mucoepidermoid Carcinoma	4	hMSH2	FE11	100%(4/4)	47.8 ± 37.8% range 10 - 89%
	Mucoepidermoid Carcinoma	4	hMLH1	G168-728	100%(4/4)	23.5 ± 16.7% range 6 - 81%
	Squamous Cell Carcinoma	1	hMSH2	FE11	100%( 1/1)	80%
	Squamous Cell Carcinoma	1	hMLH1	G168-728	100% (1/1)	25%
	Acinic Cell Adenocarcinoma	3	hMSH2	FE11	100%(3/3)	60 ± 35.6% range 20- 88%
	Acinic Cell Adenocarcinoma	3	hMLH1	G168-728	100%(3/3)	56± 42.7% range 8 - 90%
	Basal Cell Adenocarcinoma	1	hMSH2	FE11	100% (1/1)	60%
	Basal Cell Adenocarcinoma	1	hMLH1	G168-728	100% (1/1)	11%
	NOS	4	hMSH2	FE11	100%(4/4)	47.5± 32.3% range 25- 95%
	NOS	4	hMLH1	G168-728	100%(4/4)	27.5 ± 24% range 9 - 62%
	Polymorphous Low-grade Adenocarcinoma	1	hMSH2	FE11	100% (1/1)	97%
	Polymorphous Low-grade Adenocarcinoma	1	hMLH1	G168-728	100% (1/1)	16%
	Malignant Mixed Tumor	2	hMSH2	FE11	100%(2/2)	28% range 20- 36%
	Malignant Mixed Tumor	2	hMLH1	G168-728	100%(2/2)	13.5% range 12- 15%
Soares *et al.* (2018)	Sebaceous Adenocarcinoma	9	hMSH2	Polyclonal	NR	56.5% range 2- 80%
	Sebaceous Adenocarcinoma	9	hMLH1	EEPR3893	NR	62.5% range 28- 83%
	Sebaceous Adenocarcinoma associated with MTS	1	hMSH2	Polyclonal	NR	2% range 2- 80%
	Sebaceous Adenocarcinoma associated with MTS	1	hMLH1	EEPR3893	NR	28% range 28- 83%
Amaral-Silva *et al.* (2022)	Adenoid Cystic Carcinoma	19	hMSH2	Polyclonal	100% (19/19)	46.47 ± 18.06% range 8.7 - 85.6%
	Adenoid Cystic Carcinoma	19	hMSH3	Polyclonal	94.71%(18/19)	4.27 ± 5.35% range 0 - 21.3%
	Adenoid Cystic Carcinoma	19	hMSH6	Polyclonal	NR	13.90 ± 8.05% range 0.2 - 25.9%
	Mucoepidermoid Carcinoma	19	hMSH2	Polyclonal	100% (19/19)	26.87 ± 14.96% range 2.3 - 52%
	Mucoepidermoid Carcinoma	19	hMSH3	Polyclonal	NR	8.16 ± 8.52% range 0.8 - 33.6%
	Mucoepidermoid Carcinoma	19	hMSH6	Polyclonal	NR	7.72 ± 4.9% range 0.2 - 19.8%
